# Prevalence of risk factors for chronic kidney disease among adults in a university community in southern Nigeria

**DOI:** 10.11604/pamj.2015.21.120.7079

**Published:** 2015-06-13

**Authors:** Chinyere Mmanwanyi Wachukwu, Pedro Chimezie Emem-Chioma, Friday Samuel Wokoma, Richard Ishmeal Oko-Jaja

**Affiliations:** 1 Department of Medicine, Faculty of Clinical Sciences, College of Health Sciences, University of Port Harcourt, Nigeria; 2 Department of Internal Medicine, University of Port Harcourt Teaching Hospital, Port Harcourt, Nigeria

**Keywords:** Chronic kidney disease (CKD), risk factors, prevention

## Abstract

**Introduction:**

The rising prevalence of chronic kidney disease (CKD) remains a global public health challenge particularly in developing countries, including our local environment, where subjects with the disease present late and may already be in need of renal replacement therapy. Early detection of modifiable risk factors of CKD is a plausible strategy to reduce its prevalence and burden. The 2014 World Kidney Day (WKD) exercise provided a veritable opportunity to identify CKD risk factors among adult Nigerians for early intervention.

**Methods:**

Subjects were mobilized from the University community for the 2014 WKD exercise. The parameters assessed were demographics, Body mass index (BMI), blood pressures, proteinuria, glycosuria, serum creatinine and fasting plasma glucose. Glomerular Filtration Rate (GFR) was estimated using the Cockcroft-Gault equation. Data were analyzed using SPSS version 17.0.

**Results:**

A total of 259 volunteers were studied, mean age of 28.3±9.7years (16-66years). Males comprised 135(52.1%) while 124(47.9%) were females. The frequency of risk factors of CKD observed were obesity in 31(12.2%) subjects, proteinuria and glycosuria in 32(12.4%) and 7(2.7%) subjects respectively. Hypertension and hyperglycaemia were seen in 54(20.8%) and 11(4.3%) of subjects respectively. Five subjects (1.9%) had e-GFR < 60mls/min/1.73m^2^.

**Conclusion:**

Prevalence of CKD risk factors in this study population was high. There is need for continuous education, regular screening for early detection and early intervention by risk factor modification to prevent and/or reduce the growing burden of CKD and its sequelae in Nigeria.

## Introduction

Chronic kidney disease (CKD) has become a major health concern globally, especially in developing countries with a marked burden in Sub-Saharan Africa [1]. This concern is largely due to the rising prevalence of risk factors such as type 2 diabetes, hypertension, and the HIV pandemic, the enormous cost implication of its treatment, its role in cardiovascular morbidity and mortality and the fact that the disease largely afflicts the economically productive younger age groups [1, 2]. Several hospital based studies in Nigeria have put the prevalence of CKD between 1.6 - 12.4% [3] with a high prevalence of risk factors observed in various studies among different groups [4, 5]. The early stages of CKD (stages 1-3a) are generally asymptomatic; therefore, the burden of the disease at these early stages goes largely undetected and difficult to assess. The symptoms only begin to manifest when greater than fifty percent of renal functional mass has been lost [6]. Most patients therefore present late to hospital, usually in the advanced diseased states and in need of salvage dialysis. As a result therefore, regular screening to aid early detection of risk factors is paramount in the prevention of CKD and ESRD. This need cannot be over-emphasized. The aim of this study was to determine the prevalence of risk factors for CKD among adults in a University community in the South-South Region of Nigeria, for early intervention in identified cases for prevention and delay of CKD progression.

## Methods

This was a cross-sectional cohort study of adult volunteers in a University in the South-South region of Nigeria during the 2014 World kidney day exercise. Subjects for the study were mobilized through the University authorities. Verbal informed consent was obtained from each of the participants after a session of health education on kidney health. The study participants included consultant nephrologists, residents in internal medicine, house officers, medical students, and nephrology nurses, all of whom participated in data collection.

### Clinical evaluation

Demographic data were obtained through interviewer-administered questionnaires and included age, sex, family and personal history of hypertension, diabetes and kidney disease. Pregnant women and individual with acute febrile illness were excluded from the study. Weight was measured with a standard measuring scale calibrated in kilograms with subjects standing erect, bare-foot, and without heavy clothing. Height was measured with a standiometer with subjects standing feet together without shoes or head gear. Body mass index (BMI) was calculated as body weight in kilograms divided by the square of the height in meters. Obesity was defined as BMI≥ 30kg/m^2^ according to the WHO guidelines [7]. Blood pressure was measured with a standard (Accosson) mercury sphygmomanometer on the patients’ right arm in the seated position with feet on the floor after at least a five-minute rest. Systolic and diastolic blood pressures were taken at Korotkoff phases 1 and 5 respectively to the nearest 2 mmHg. The average of two blood pressure measurements taken five minutes apart was used. Hypertension was defined as SBP ≥ 140mmHg and /or DBP ≥ 90mmHg [8]. Elevated SBP was defined as SBP ≥ 140mmHg and elevated DBP as DBP ≥ 90mmHg.

### Biochemical analysis

A sample of five millimetres (5mls) of venous blood was taken from each subject for assessment of serum creatinine. The samples were placed in lithium heparin bottles (gently mixed) and transported immediately to the chemical pathology laboratory. Proteinuria and glycosuria were assessed in subjects’ urine using combi-2 dipstick. Proteinuria and glycosuria were defined as the presence of at least 1+ of protein and 1+ of glucose on dipstick respectively. Random blood sugar was measured using the Accucheck Glucometer, and results were expressed in mmol/l. Hyperglycaemia was defined as random blood sugar > 11.1mmol/l (>200mg/dl). The Glomerular filtration rate was estimated using the Cockcroft-Gault equation [9] with correction for body surface area (BSA). Body surface area was calculated using the DuBois and DuBois formula [10].

### Statistical analysis

Collated data were analysed using statistical package for social sciences (SPSS) version 17.0 analytic software. Continuous variables were compared with the independent samples T-test and the Chi-square test was used to compare categorical variables. Pearson correlation was used to analyse the relationship between variables.

## Results

### Demographic and clinical characteristics of subjects

A total of 259 subjects were screened for CKD in this study. The mean age of subjects in the study was 28.3±9.7 years with a range of 16 to 66years. Majority of the study population 235(90.7%) were less than 45 years, 23(8.9%) were between 45 years and 65 years. Only one subject (0.4%) was over 65 years. There were more males than females, 135 (52.1%) and 124 (47.9%) respectively. Personal history of hypertension, diabetes and CKD was obtained in 10(3.9%), 5(1.9%) and 4(1.5%) subjects respectively. Family history of hypertension and diabetes was obtained in 87(33.6%) and 66(25.5%) of subjects respectively and only 4(1.5%) subjects had a family history of CKD.

### Clinical characteristics of study group

The weight in kilograms was significantly higher among the male subjects, though no statistical significant difference was observed in the BMI among the male and female subjects. The systolic and diastolic blood pressures were higher the male population, with a lower eGFR observed among the female population. These results were statistically significant. The clinical characteristics of the study group are shown in Table 1.

**Table 1:** Clinical and biochemical characteristics of study population

Variable	Total population Mean±SD n= 259	Males Mean±SD n= 135	Females Mean±SD n= 124	p value
Weight (kg)	68.9±14.2	73.6±13.1	63.8±13.3	0.000
BMI (kg/m2)	24.7±4.5	24.7±3.9	24.6±5.0	0.794
SBP (mmHg)	117.3±15.5	121.3±15.8	113.1±14.0	0.000
DBP (mmHg)	75.7±11.7	78.3±11.5	72.9±11.3	0.000
eGFR (mls/min)	108.9±29.7	114.9±30.8	102.6±27.2	0.001
RBG (mmol/l)	5.56±1.8	5.7±2.4	5.3±0.7	0.114

### Frequency of risk factors for CKD

**Figure 1:**
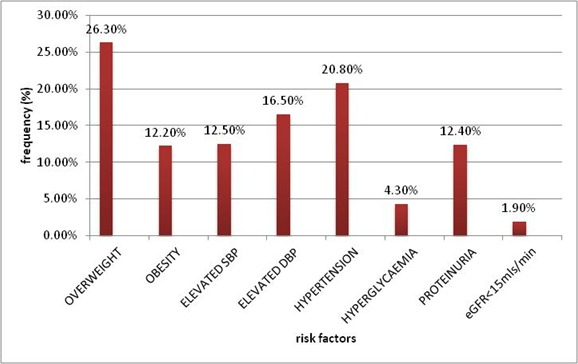
Frequency of risk factors for CKD among the study population

Hypertension was observed in 20.8% of subjects, 12.2% of subjects were obese and 4.3% had hyperglycaemia. The eGFR was less than 60mls/min/1.73m^2^ in 1.9% of subjects. The frequency of risk factors for CKD are shown in Figure 1. There was a significant negative correlation between age of subjects and eGFR but no significant association was observed between eGFR and either systolic or diastolic blood pressures. This is as shown in Table 2.

**Table 2:** Correlation of eGFR with clinical parameters

Variable	Correlation coefficient (r)	p-value
Age(years)	-0.231	<0.001
BMI(kg/m2	0.511	<0.001
SBP(mmHg)	0.005	0.936
DBP(mmHg)	0.009	0.891

## Discussion

The mission of the world kidney day is to raise global awareness of the importance of the kidneys to overall health, [11] thereby reducing the frequency of CKD and its attendant burden. Population-based education and screening programmes, as were carried out in this study, are intended to increase the rates at which subjects with kidney disease will be detected, enabling further evaluation of those identified and permitting disease modifying interventions to be instituted [12]. The risk factors of CKD screened for in this population included hypertension, obesity, hyperglycaemia, proteinuria, glycosuria as well as eGFR. The prevalence of hypertension observed in this study was 20.8%; this falls within the range of the overall prevalence of hypertension in Nigeria reported to be between 8.0- 46.4% [13]. Ordinioha [14] reported a similar prevalence of 21.3% among lecturers in Port Harcourt. Similarly a report among civil servants in Kano [15] showed a prevalence of 29.8%. Hypertension remains among the top three causes of CKD in Nigeria [16, 17] and is also a common cause of CKD in other parts of Sub-Saharan Africa [1] and when uncontrolled, it is known to hasten the progress of CKD as well as increase cardiovascular complications. This relatively high prevalence of hypertension may be associated with increasing urbanization, and adoption of western lifestyle in this environment. According to the World Health Organisation (WHO), in 2014, more than 1.9billion persons worldwide were overweight and of these greater than 600 million were obese. [18] Sub-Saharan Africa is not exempt from the obesity epidemic and Abubakari et al [19] in a review reported an obesity prevalence of 10% among West African adults. In this study, the prevalence of overweight and obesity was comparable to that earlier reported in a systematic review among adult Nigerians [20]. This relatively high prevalence of overweight and obesity observed in this study can be attributed to increased popularity in this environment of western diets rich in sugar, saturated fats and refined carbohydrates.

There is also an apparent upsurge in the consumption of fast foods; this with sedentary lifestyles may encourage the development of obesity. The commonest risk factors for CKD remain diabetes and hypertension [16, 17] and these conditions are known to be strongly associated with obesity. Obesity has been shown to have effects independently impacting on renal haemodynamics and its tendency to cause and/or increase proteinuria may account for its strong association with worsening progression of CKD [21, 22]. Hyperglycaemia in this study was observed in 4.3% of the participants, comparable to that observed in other studies. [4, 5] In Port Harcourt, Nigeria, the prevalence of diabetes was reported to be 6.8% among adults [23] and Nigeria is also said to have the largest number of people with type 2 diabetes mellitus in Africa [24]. Diabetes is also known to be the leading cause of CKD worldwide [25] and is gaining an increasing important role as a cause of CKD and ESRD in Sub-Saharan Africa [1] and in Nigeria [26], therefore this prevalence observed here poses a cause for concern. The prevalence of proteinuria in this study was 12.4%. A higher prevalence of 19.4% was reported among civil servants in Kano [15], while a lower prevalence of 5.6% was observed among civil servants in Bayelsa. [4] This high prevalence observed in this study is perturbing in this unselected apparently healthy population of young people. Proteinuria is an independent risk factor and an early indicator of kidney disease and its persistence is known to be associated with progression of kidney disease [27]. The prevalence of CKD (eGFR< 60mls/min/1.73m^2^) 1.9% in this cohort was low compared to recent reports in Nigeria [4, 28]. This is due to the relative young study population. Secondly the study is among a cohort of University population who are predominantly healthy. It is also well known that the eGFR declines with increasing age. This study also showed a significant negative correlation between age and eGFR. Action taken on subjects in whom risk factors of CKD were detected: these subjects were counselled and referred to the University Health Centre for further follow up. This study may have been limited by its cross-sectional nature (as the participants were not screened again to determine if there will be persistence of these risk factors), the probable narrow social strata of the study population and the method employed for assessing proteinuria.

## Conclusion

Though the prevalence of e-GFR
